# *femaleless* Controls Sex Determination and Dosage Compensation Pathways in Females of *Anopheles* Mosquitoes

**DOI:** 10.1016/j.cub.2020.12.014

**Published:** 2021-03-08

**Authors:** Elzbieta Krzywinska, Luca Ferretti, Jianwei Li, Jian-Chiuan Li, Chun-Hong Chen, Jaroslaw Krzywinski

**Affiliations:** 1 The Pirbright Institute, Ash Road, Pirbright, Surrey GU24 0NF, UK; 2 Big Data Institute, Nuffield Department of Medicine, University of Oxford, Old Road Campus, Oxford OX3 7LF, UK; 3 National Institute of Infectious Diseases and Vaccinology, National Health Research Institutes, 35 Keyan Road, Zhunan, Miaoli 350401, Taiwan; 4 National Mosquito-Borne Disease Control Research Center, National Health Research Institutes, 35 Keyan Road, Zhunan, Miaoli 350401, Taiwan

**Keywords:** *Anopheles gambiae*, transgenesis, sex determination pathway evolution, female-specific lethality, malaria vectors, genetic vector control

## Abstract

The insect sex determination and the intimately linked dosage compensation pathways represent a challenging evolutionary puzzle that has been solved only in *Drosophila melanogaster*. Analyses of orthologs of the *Drosophila* genes identified in non-drosophilid taxa^1^^,^^2^ revealed that evolution of sex determination pathways is consistent with a bottom-up mode,^3^ where only the terminal genes within the pathway are well conserved. *doublesex* (*dsx*), occupying a bottom-most position and encoding sex-specific proteins orchestrating downstream sexual differentiation processes, is an ancient sex-determining gene present in all studied species.^2^^,^^4^^,^^5^ With the exception of lepidopterans, its female-specific splicing is known to be regulated by *transformer* (*tra*) and its co-factor *transformer-2* (*tra2*).^6^, ^7^, ^8^, ^9^, ^10^, ^11^, ^12^, ^13^, ^14^, ^15^, ^16^, ^17^, ^18^, ^19^, ^20^ Here we show that in the African malaria mosquito *Anopheles gambiae*, a gene, which likely arose in the *Anopheles* lineage and which we call *femaleless* (*fle*), controls sex determination in females by regulating splicing of *dsx* and *fruitless* (*fru*; another terminal gene within a branch of the sex determination pathway). Moreover, *fle* represents a novel molecular link between the sex determination and dosage compensation pathways. It is necessary to suppress activation of dosage compensation in females, as demonstrated by the significant upregulation of the female X chromosome genes and a correlated female-specific lethality, but no negative effect on males, in response to *fle* knockdown. This unexpected property, combined with a high level of conservation in sequence and function in anopheline mosquitoes, makes *fle* an excellent target for genetic control of all major vectors of human malaria.

## Highlights

• *fle* is a new sex determination pathway element conserved in *Anopheles* mosquitoes
• *fle* may have originated in the *Anopheles* lineage and is highly conserved in *Anopheles*
• *fle* suppresses activation of dosage compensation in females
• Depletion of *fle* transcripts is lethal or otherwise deleterious to females

## Teaser

Krzywinska et al. identify a new element of the sex determination pathway in *Anopheles*. *femaleless* (*fle*), in addition to controlling splicing of *dsx* and *fru*, is essential for suppression of dosage compensation and viability of females. Female-deleterious effects upon *fle* knockdown make *fle* a promising target for control of malaria mosquitoes.

## Results and Discussion

### *fle* Is a Sex Determination Pathway Component in *Anopheles*

**Figure 1:**
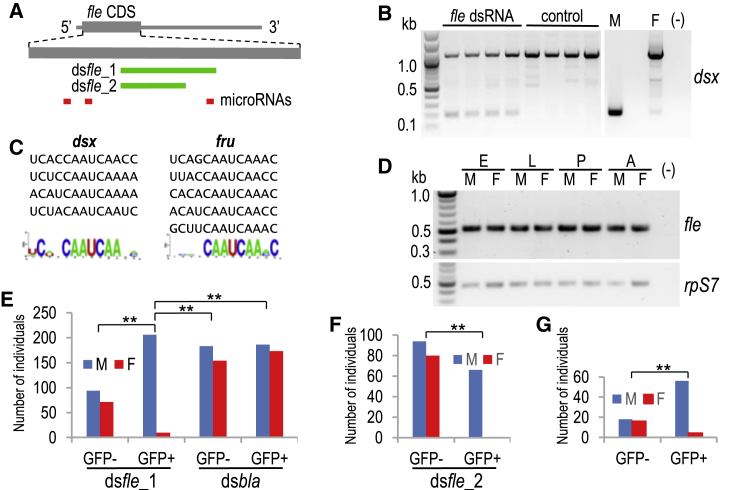
*fle* Is a Sex Determination Gene Necessary for *dsx* Splicing and Survival in *Anopheles* Females (A) A schematic of the *A. gambiae fle* transcript, with the location of RNAi targets within the coding sequence. (B) RT-PCR analysis of the *dsx* splicing pattern in *A. gambiae* Sua5.1 cells transfected with *in vitro*-synthesized *fle*_1 dsRNA, as compared with control non-transfected cells. (C) Sequences of the putative Fle binding sites in the *A. gambiae dsx* and *fru* female-specific exons. Presented are 13-nt fragments with similarity to the *Drosophila* repeat elements [TC(T/A)(T/A)CAATCAACA]. (D) RT-PCR analysis of *fle* transcription during *A. gambiae* development. E, embryos; L, larvae; P, pupae; A, adults; (−), negative control; M, male; F, female. Ribosomal protein S7 (rpS7) transcript levels were used as a gel loading control. (E and F) Knockdown of *fle* expression causes female-specific lethality in *A. gambiae* embryos. A summary of three independent microinjection experiments using *fle*_1 (E) and *fle_*2 (F) dsRNA fragments, with *bla* dsRNA used as control. GFP−/+ denote cohorts of individuals scored during the first larval instar as GFP-negative or GFP-positive. ^∗∗^p < 0.0001; Fisher’s exact test. (G) Knockdown of *fle* ortholog expression causes female-specific lethality in *A. stephensi* embryos. A summary of two independent *fle* dsRNA microinjection experiments. GFP−/+ as in (E). ^∗∗^p < 0.0001; Fisher’s exact test. See also Figures S1–S3.

In the mosquitoes from the genus *Anopheles*, sex is chromosomally determined, with the XX individuals developing as females and the XY individuals developing into males. Three components from the molecular pathway controlling this process have been identified in the African malaria mosquito *Anopheles gambiae*: a Y-chromosome-linked primary signal gene, *Yob*, conferring maleness^21^^,^^22^ and, on the opposite end, *dsx*^23^ and *fru*.^24^ To search for molecules transducing the sex-determining instruction, we queried the *A. gambiae* genome with the *D. melanogaster* Tra2 sequence using a BLAST algorithm. Tra2 is an RNA-binding protein with a characteristic structure consisting of an RNA recognition motif (RRM) flanked by two arginine-rich/serine-rich (RS) domains containing multiple serine-arginine dipeptides.^25^ In all significant BLAST hits, sequence similarity was limited to the RRM region. The top two hits, AGAP029421 (e value = 1e−28) and AGAP006798 (e value = 1e−25), encoding proteins with a structure typical of Tra2, are regarded here as the *tra2* homologs, named, respectively, *tra2b* and *tra2a*. To assess whether they are involved in sex determination, we investigated the effect of their transient knockdown on *dsx* splicing in the *A. gambiae* Sua5.1 female-like cell line. Transfection experiments using the *in-vitro*-synthesized double-stranded RNA (dsRNA) of either *tra2a* or *tra2b* did not yield the expected change in the *dsx* splicing pattern from the female to the male mode, indicating that neither has the sex-determining role (Figures S1A and S1B), consistent with the findings that *tra2* homologs have no role in *dsx* splicing in a culicine mosquito, *Aedes albopictus*.^26^ In contrast, knockdown of the third-best hit, AGAP013051 (e value = 5e−18), caused a clear decrease of the female, and gain of the male, *dsx* transcript isoforms, as compared with the control non-transfected Sua5.1 cells (Figures 1A, 1B, and S1C). This result, further validated in transgenic *A. gambiae* lines with a stable AGAP013051 knockdown (see below), demonstrated that AGAP013051 represents a sex determination pathway element regulating *dsx* splicing in *A. gambiae* females.

Apart from similarity within the RRM, AGAP013051 substantially differs from insect *tra2* orthologs in the length of the coding region (420 amino acids compared to 232–299 amino acids in the described Tra2 proteins)^10^^,^^11^^,^^14^^,^^16^^,^^27^, ^28^, ^29^, ^30^ and structure of the encoded protein, including lack of typical RS domains and presence of two additional putative RRM regions in the N-terminal half of the protein (Figures S2A and S2B). Combined with the results of a phylogenetic analysis, in which AGAP013051 does not cluster with Tra2 proteins but is grouped with a putative splicing factor, *Nix*, functioning as the male determiner in *Aedes aegypti*^31^ (Figures S2C and S2D), the above indicates that AGAP013051 and *tra2* may be only distantly related. We named the gene *femaleless* (*fle*), to reflect the associated knockdown phenotypes described below.

The RRM is a highly abundant motif in various eukaryotic proteins. It folds into a β_1_α_1_β_2_β_3_α_2_β_4_ sandwich topology, with the β_1_ and β_3_ sheets encompassing, respectively, ribonucleoprotein 2 (RNP2) and RNP1 sequence elements that are required for specific binding to the RNA sequences.^25^^,^^32^^,^^33^ In *Drosophila*, Tra2 molecules bind six 13-nt repeat elements [UC(U/A)(U/A)CAAUCAACA], clustered on the *dsx* pre-mRNA within the untranslated region (UTR) of the female-specific *dsx* exon 4, to promote the use of an adjacent upstream weak splice acceptor site, leading to splicing of *dsx* into the female form.^8^^,^^34^^,^^35^ *fle* may directly target a (U/A)C(U/A)(U/C/A)CAAUCAA(U/C/A)(C/A) sequence, which forms a four-repeat cluster within the UTR of the *A. gambiae* female-specific *dsx* exon 5 (Figure 1C). These putative *fle* targets map to regions of very low nucleotide diversity in natural *A. gambiae* populations^36^ and coincide with blocks of increased conserved sequence across *Anopheles* species (data not shown), which lends support to the notion of their functional significance. The *A. gambiae fru* gene, whose sex-specific splicing is also *fle* dependent (this study; see below), contains three putative *fle* binding sites clustered at the 5' end of the female-specific exon 3 and two binding sites in the sex-specifically processed exon 4. Their consensus sequence shares the same invariant core (CAATCAA) with the *A. gambiae dsx* repeat elements, as well as with the counterparts from the *Drosophila dsx* and *fru* (Figure 1C). Considerable variation within the nucleotide positions flanking the core suggests that only the core may be important for efficient *fle* binding.

We queried the NCBI whole-genome shotgun contigs database with the *fle* amino acid sequence using tBLASTn to evaluate the phylogenetic distribution of the *fle* orthologs. Significant hits were identified only in the genomes of genus *Anopheles* representatives, with highly conserved 1-to-1 orthologs detected in each case (Figure S3A), indicating that *fle* has been subject to strong functional constraints throughout approximately 100 million years of *Anopheles* lineage evolution.^37^^,^^38^ A lack of discernible orthologs beyond anopheline genomes suggests that *fle* may have originated in a recent ancestor of the *Anopheles* lineage. To test whether *fle* could have arisen through a gene duplication, we searched the *A. gambiae* annotated proteins for sequences similar to Fle. The top hit was to AGAP001643 (the gene restricted to the family Culicidae [mosquitoes]), which at the amino acid level shares a significant similarity (BLASTp, e value = 4e−20) to the N-terminal half of Fle and a lower similarity to the C-terminal RRM (Figures S3B and S3C). The first intron in AGAP001643 and the intron in *fle* split a codon located at the putative homologous alignment position, further suggesting a common origin of the two genes. Thus, *fle* may represent an ancient paralog of AGAP001643, which after a gene duplication rapidly diverged to assume essential developmental functions. In that case, the similarity between Tra2 and Fle within the RRM could represent a case of convergent evolution driven by the adaptation of *fle* to efficiently bind highly conserved target sequences. Alternatively, *fle* could have originated from a fusion between an AGAP001643 copy and a *tra2* copy, followed by subsequent rearrangements. A likely evolutionary scenario cannot be reliably inferred because the sequences in question are highly divergent.

### Transient *fle* Knockdown Kills *Anopheles* Female Embryos

The *fle* gene produces a single transcript and is constitutively expressed throughout development in both *A. gambiae* sexes (Figure 1D). To assess whether a transient *fle* knockdown has an effect on *A. gambiae* early development, we injected the *fle* dsRNA into non-sexed preblastoderm embryos using an established protocol.^21^ The injection mix contained a control plasmid with a green fluorescent protein (GFP) expression cassette, which allowed for an easy identification of individuals that received sufficient amounts of nucleic acids. Surviving first-instar larvae were sorted into GFP-negative and GFP-positive cohorts, and at the pupal stage mosquitoes were sexed. A very strong male bias was observed among the GFP-positive mosquitoes (Figures 1E and 1F), whereas in the GFP-negative group and in the control group of *A. gambiae* embryos injected with heterologous *bla* dsRNA the sex ratio was not significantly biased. A random sample of individuals (n = 20) from the strong GFP cohort were sexed using PCR^21^ and confirmed to have the XY karyotype (data not shown). Similar results, with a strongly male-biased survival, were obtained after microinjection of *Anopheles stephensi fle* dsRNA into embryos of *A. stephensi* (Figure 1G), a species that diverged from the *A. gambiae* lineage over 40 million years ago (mya).^37^^,^^39^ These results indicated that embryonic knockdown of *fle* is lethal to genetic females but apparently has no discernible effect on the development of genetic males. The female death was occurring during the embryo stage, because the numbers of hatched GFP-positive larvae were comparable to the numbers of pupating individuals.

### *fle* Links the Sex Determination and Dosage Compensation Pathways

**Figure 2:**
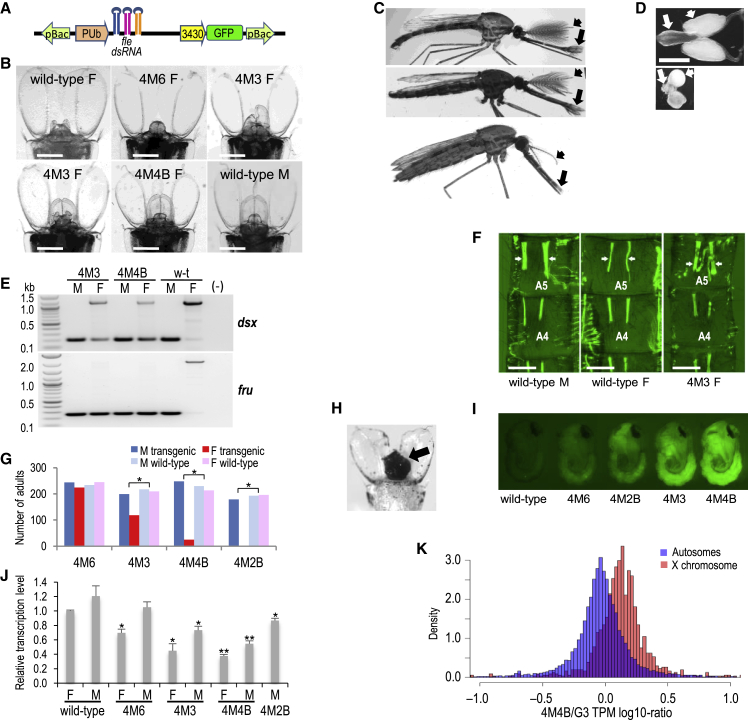
Stable *fle* Knockdown in *A. gambiae* Causes Female Masculinization and Death through Abnormal Upregulation of Transcription from the X Chromosome (A) Schematic representation of the transgenic *fle* knockdown construct. (B) Pupal abdominal termini of the wild-type and *fle* knockdown transgenic mosquito lines. Scale bars, 200 μm. (C) Adults. From top: wild-type male, 4M4B line female, wild-type female. Note male-like antennae (short arrows) and palps (long arrows), and a slender abdomen with a male-like uninverted copulatory apparatus. (D) Reproductive system: ejaculatory pump (long white arrows) and accessory glands (short white arrows) in a wild-type male and 4M4B female. Scale bar, 100 μm. (E) RT-PCR analysis of *dsx* and *fru* splicing patterns in females from selected transgenic lines. (F) A sexually dimorphic pair of dorsal muscles (indicated by white arrows) in the fifth segment of the abdomen (A5), likely homologous to the *Drosophila* muscle of Lawrence, is of similar thickness in wild-type males and transgenic females, and considerably thicker than in wild-type females. Scale bars, 200 μm. (G) Proportions of individuals reaching adulthood in selected transgenic lines. The lines have been maintained by backcrossing of transgenic males with the wild-type line females at every generation, which should yield 50% of wild-type progeny and equal proportions of the sexes in the transgenic and wild-type groups. However, apart from line 4M6, a highly significant deficiency of transgenic females was observed (^∗^p < 0.0001; chi-square test). In line 4M2B, no transgenic females developed beyond the embryonic stage. (H) Pupal abdominal terminus with a characteristic black tumor (arrow) in a 4M4B female. (I) Fluorescent marker expression in selected transgenic lines. (J) Levels of *fle* transcripts in the pupae of the wild-type line G3 and in selected transgenic lines quantified by qRT-PCR and normalized relative to the *fle*/*rpS7* transcription level in wild-type females. The error bars represent standard deviations. ^∗^p < 0.01, ^∗∗^p < 0.001; Student’s t test. (K) Comparison of transcription levels from the autosomes and the X chromosomes in transgenic line 4M4B and wild-type line G3 females. Shown are transcripts per kilobase million (TPM) ratios. The median expression ratio between the 4M4B and G3 lines for the autosomes (0.92) and for the X chromosomes (1.34) indicates that the X chromosome genes in the transgenic line females are significantly overexpressed; p < 10^−15^; Mann-Whitney U test. A similar trend for the median shrunken fold change for the autosomal (0.985) and for the X linked (1.22) transcripts confirms that the effect is statistically robust. See also Table S1.

Using piggyBac-based transgenesis, we generated six *A. gambiae* lines that stably produce a polycistronic transcript encoding three microRNAs designed to silence expression of *fle* (Figure 2A). In each line, *fle* knockdown affected the development of females, with phenotypic effects defined by the genomic location of the transgene insertions (none known to interrupt a gene; Table S1). The abnormalities in sexually dimorphic characters ranged from mild, such as asymmetric development or loss of cerci (terminal appendages of the female abdomen) in line 4M6, to an extensive masculinization manifested in the development of a nearly normal male copulatory apparatus and male-like head appendages in line 4M4B (Figures 2B and 2C). The internal reproductive system was similarly affected, including underdeveloped or atrophied ovaries, atrophied spermatheca, partially developed male accessory glands, and a rudimentary ejaculatory pump (Figure 2D). The level of masculinization of transgenic females was correlated with a substantially altered splicing of *dsx* (similar to the effects of *fle* knockdown in Sua5.1 cells) and, additionally, *fru* (Figure 2E), whose sex-specific splicing is conserved between *Anopheles* and *Drosophila*.^24^ In *Drosophila*, female-specific *fru* transcripts do not code for a functional protein, whereas the male-specific isoforms encode a protein produced in a small subset of neurons in the central nervous system, where it regulates male sexual behavior^40^ and specifies the development of a male-specific muscle of Lawrence (MOL) in the fifth abdominal segment.^41^ The *fru* ortholog apparently performs the same function in *A. gambiae*.^24^ Consistent with that notion, we found a male-like, bilaterally paired muscle reminiscent of MOL in the fifth abdominal segment of the *A. gambiae* transgenic females (Figure 2F). Moreover, in none of the lines were transgenic females attracted to a blood source, likely because they produce the male form of *fru*.

Beyond masculinization, *fle* depletion caused partial or complete female lethality in some lines (Figure 2G). In line 4M2B, only one-third of hatched larvae at each generation were transgenic. They developed exclusively as males, which indicated that transgenic females were dying during embryonic stage, consistent with the female-specific embryonic lethality in transient *fle* knockdown experiments. In line 4M4B, female death was occurring during late developmental stages; a small proportion of individuals that survived to adulthood died shortly (up to 3 days) after eclosion. The majority of females dying as pupae developed a distinctive black tumor at the abdominal terminus (Figure 2H). In contrast to females, males appeared not to be affected in any of the transgenic lines.

The severity of the aberrant female development in transgenic lines was roughly correlated with the level of expression of the GFP transformation marker (Figure 2I) and with the extent of the *fle* knockdown (Figure 2J) in pupae. Line 4M2B, with an invariable female embryonic-lethal phenotype but with a relatively low GFP expression and a low *fle* knockdown level in male pupae, represented a notable exception, suggesting an involvement of an embryonic enhancer driving higher microRNA expression and a resulting increased *fle* knockdown during early development. Overall, the observed levels of knockdown were surprisingly low, even in a line exhibiting strong masculinization (Figure 2J), suggesting that *fle* is a haploinsufficient gene. Presumably, *fle* knockdown below a certain critical threshold leads to female lethality.

Sex-specific lethality caused by loss-of-function mutations or knockdown of sex determination genes results from misregulation of dosage compensation in *Drosophila* and a silk moth, *Bombyx mori*.^19^^,^^42^, ^43^, ^44^ In *Drosophila*, dosage compensation relies on a two-fold upregulation of transcription from the single male X chromosome to the levels of expression from both X chromosomes in females.^45^ Dosage compensation machinery is not assembled in *Drosophila* females, because SXL, a female-specific protein involved in sex determination, prevents translation of MSL-2, a key protein of the dosage compensation complex.^46^ Mutations in *Sxl*, or in genes involved in *Sxl* regulation, lead to overexpression of X linked genes and female death during embryogenesis.^42^^,^^43^^,^^47^^,^^48^ In *A. gambiae*, dosage compensation also operates by upregulation of the X chromosome in males and that process is controlled by the primary sex determiner gene *Yob*.^21^^,^^49^ Similarly, in *A. stephensi*, the X chromosome dosage in males is regulated by the Y linked maleness gene *guy-1*.^50^ Female-specific lethality observed in this study suggests that dosage compensation is activated in *Anopheles* females in response to depletion of *fle* transcripts. To evaluate whether misregulation of the X chromosome transcription is indeed involved, we compared transcriptomes of female pupae from a wild-type line and from the 4M4B transgenic line, in which female lethal effects occur during late stages of development. Relative to the autosomes, transcription from the X chromosomes in transgenic females was significantly upregulated (Mann-Whitney U test, p < 10^−15^), with an overall transcription increase by more than 40% (Figure 2K), which is apparently toxic and leads to preimaginal death. The X-chromosome-wide overtranscription caused by misregulation of dosage compensation mechanisms mimics X chromosome aneuploidy, and is known to lead to tumorigenesis in *Drosophila* and cancers in mice,^51^^,^^52^ and apparently is the cause of abdominal tumors developing in the 4M4B female pupae (Figure 2G). However, tumors in the genital area were also observed in a moth, *Plutella xylostella*, female mutants with disrupted *dsxF* transcripts.^53^

**Figure 3:**
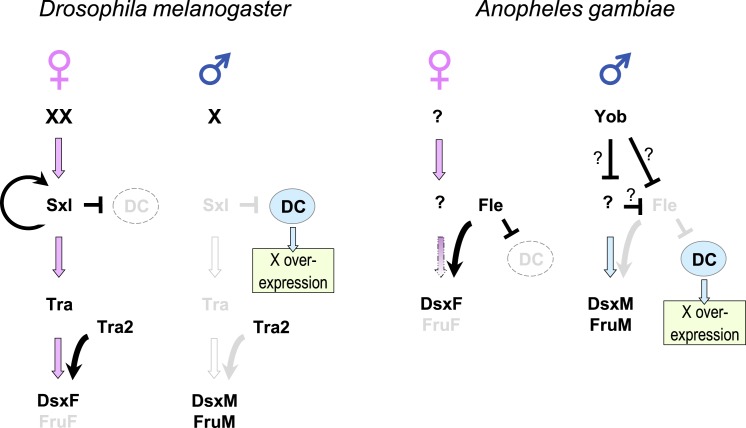
Proteins Determining Sex in Somatic Tissues of *D. melanogaster* and *A. gambiae* In fruit fly females, a double dose of X linked elements activates productive transcription of *Sxl*, leading to establishment of a positive feedback loop of Sxl production. Sxl controls splicing of *tra* into a productive form, which, together with Tra2, is necessary for splicing of *dsx* into a productive form and *fru* into a non-productive form. Sxl also prevents assembly of the dosage compensation (DC) complex by blocking translation of *msl2*, a critical component of the complex. In fruit fly males, a single dose of X linked proteins is insufficient to initiate a productive transcription of *Sxl*. As a result, dosage compensation is activated, *tra* is spliced into a non-productive form, and the default splicing of *dsx* and *fru* productive male forms occurs. In *Anopheles* females, Fle is necessary for the splicing of *dsx* and *fru* into productive and non-productive forms, respectively, as well as for repression of dosage compensation. The upstream sex-determining molecules remain to be identified; it is unclear whether Fle requires a cofactor to promote splicing of *dsx* and *fru*. In males, the primary sex determiner *Yob* triggers the sex determination pathway and inactivates Fle by a yet unknown mechanism, allowing activation of dosage compensation. Fle does not take part in the splicing of *dsx* or *fru* into male forms.

Control of the *dsx* and *fru* female-specific splicing by a non-sex-specifically expressed *fle* provokes a question about the mechanism of that process. In most studied holometabolous insects, the female specificity of *dsx* splicing relies on Tra, which is produced only in females, and whose interaction with Tra2 via RS domains is necessary to stabilize the spliceosome assembly on the weak acceptor splice site.^8^^,^^9^^,^^11^^,^^14^^,^^16^^,^^25^ Considering an apparent absence of *tra* in the *A. gambiae* genome,^2^^,^^54^ as well as a larger size and a structural dissimilarity of Fle as compared to Tra2, it is conceivable that Fle does not have an obligatory Tra-like partner and that binding of Fle alone to its putative targets (Figure 1C) is sufficient to promote the spliceosome assembly in females, whereas in males that process is interrupted by Yob (Figure 3).

In summary, in addition to a vital role in the splicing of *dsx* and *fru* into a female form, *fle* represses dosage compensation in *A. gambiae* females through a yet unknown mechanism. As such, *fle* is only the second known, after *Sxl* in *Drosophila*, molecular link between the sex determination and dosage compensation pathways in insects (Figure 3). Female lethality in response to *fle* knockdown indicates that dosage compensation is regulated by *fle* also in *A. stephensi*.

### *fle* as a Target Molecule for *Anopheles* Control

Mosquitoes from the genus *Anopheles* are the exclusive vectors of *Plasmodium* parasites that cause human malaria, a disease infecting annually nearly 230 million people and causing over 400,000 deaths worldwide.^55^ Over 90% of malaria cases occur in sub-Saharan Africa, where *A. gambiae* is the primary vector. Control of the disease relies heavily on the use of insecticides, which are increasingly ineffective because of the emergence and spread of insecticide resistance in natural *Anopheles* populations.^55^^,^^56^ Various genetic control approaches, proposed to complement the existing insecticidal tools,^57^^,^^58^ rely on mass releases of irradiated or otherwise genetically modified males, which through mating with wild-type females spread desirable traits, such as sterility, female lethality, or inability to transmit pathogens, to cause mosquito population suppression or modification. The requirement for male-only releases is dictated by ethical and safety considerations, because only females feed on blood, and released modified females could contribute to biting and parasite transmission. To date, maleness genes *Yob* and *guy-1* have been found to have female-killing properties,^21^^,^^59^ and thus could be used to *conditionally* eliminate females of the *A. gambiae* species complex and *A. stephensi*, respectively, in genetic control operations.^58^ This study, in addition to advancing our understanding of sex determination and dosage compensation regulation in insects, identifies *fle* as a universal molecule, conserved in sequence and function in anopheline mosquitoes, that could be targeted in genetic control to eliminate females of all major malaria vector species. The apparent haploinsufficiency makes *fle* unsuitable as a target of homing gene drives; however, female-specific deleterious effects could be exploited in conditional *fle* knockdown transgenic lines to suppress populations of various *Anopheles* vector species.

## STAR★Methods

### Key Resources Table

**Table:** 

REAGENT or RESOURCE	SOURCE	IDENTIFIER
**Bacterial and Virus Strains**		

*E. coli* DH10B ElectroMax	Thermo Fisher Scientific	Cat#18290015

**Chemicals, Peptides, and Recombinant Proteins**		

Lipofectamine 2000 Transfection Reagent	Invitrogen	Cat#11668019
Schneider’s Modified Medium	Lonza	Cat#04-351Q

**Critical Commercial Assays**		

PureLink RNA Micro Kit	Invitrogen	Cat#12183016
ActinGreen 488 ReadyProbes Reagent	Thermofisher Scientific	Cat#R37110
LunaScript RT SuperMix Kit	New England Biolabs	Cat#E3010S
Luna Universal qPCR Master Mix	New England Biolabs	Cat#M3003S
DNeasy Blood & Tissue Kit	QIAGEN	Cat#69504
MEGAscript RNAi T7 Kit	Life Technologies	Cat#AM1626
One-Step RT-PCR System	Invitrogen	Cat#12574026

**Deposited Data**		

RNA-Seq data	This study	ENA: PRJEB38605

**Experimental Models: Cell Lines**		

*A. gambiae* Sua5.1	^60^	N/A

**Experimental Models: Organisms/Strains**		

*A. gambiae* G3 strain	BEI Resources	MRA-112
*A. stephensi* AF-SDA500 strain	Infravec2	V.2.3.1.L.FR.1.0

**Oligonucleotides**		

See Table S2 for detailed information on used oligonucleotides	This study	N/A

**Recombinant DNA**		

p165	^61^	GenBank: KU189142
pUAST-attB	^62^	GenBank: EF362409
pBac_pattPs_fle_3miR	This study	GenBank: MW147152
pENTR R4-vas2-Transposase-R3	^63^	N/A
pGEM-T Easy Vector System	Promega	Cat#A1360

**Software and Algorithms**		

BLAST	^64^	https://blast.ncbi.nlm.nih.gov/Blast.cgi
Jpred-4	^65^	http://www.compbio.dundee.ac.uk/jpred/
ClustalX2	^66^	https://clustalx.software.informer.com/2.1/
MEGA v. 7.0.26	^67^	https://www.megasoftware.net/
WebLogo	^68^	https://weblogo.berkeley.edu/logo.cgi
ImageJ	^69^	https://imagej.nih.gov/ij/
Kallisto v0.46	^70^	https://pachterlab.github.io/kallisto/
DESeq2	^71^	http://bioconductor.org/packages/release/bioc/html/DESeq2.html
apeGLM	^72^	https://bioconductor.org/packages/release/bioc/html/apeglm.html

**Other**		

QuantStudio 3 Real-Time PCR System	Applied Biosystems	Cat#A28136

### RESOURCE AVAILABILITY

#### Lead Contact

Further information and requests for resources and reagents should be directed to and will be fulfilled by the Lead Contact, Jaroslaw Krzywinski (jaroslaw.krzywinski@pirbright.ac.uk).

#### Materials Availability

All relevant data supporting the key findings of this study are available within the article and its Supplementary Information files or from the corresponding author upon a reasonable request.

#### Data and Code Availability

The RNA-seq data generated during this study are available at the European Nucleotide Archive with the accession number PRJEB38605.

### EXPERIMENTAL MODEL AND SUBJECT DETAILS

#### Mosquito strains

*A. gambiae* G3 strain, transgenic lines generated on the *A. gambiae* G3 strain background, and *A. stephensi* AF-SDA500 strain were reared at 28°C and 80% humidity on 12 h: 12 h light:dark cycle, following the standard protocol.^73^ Larvae were reared in plastic trays filled with 1 L of deionised water and provided with ground TetraMin tropical fish food flakes (Tetra). The adults were kept in BugDorm-1 (30 × 30 × 30 cm) or BugDorm-4 (24.5 × 24.5 × 24.5 cm) cages (BugDorm) and provided with 10% sucrose solution *ad libitum*. A 1:1 mixture of time-expired human red blood cells and plasma sourced from a blood bank was used for feeding females through the Hemotek membrane feeder.

#### Mosquito cells

The *A. gambiae* female-like Sua5.1 cells^60^ were cultured at 28°C in Schneider’s Modified medium (Lonza) supplemented with 10% fetal bovine serum (PAA), and 100 U/ml Penicillin and 100 μg/ml Streptomycin (Life Technologies).

### METHOD DETAILS

#### Sequence analyses

All similarity searches were conducted using BLAST,^64^ with the word size = 2. The search for the *A. gambiae dsx* splicing factors within the *A. gambiae* PEST strain genome was conducted using a BLASTp and tBLASTn algorithm, with the expect value 1e-7, and the *D. melanogaster* Tra2 (FlyBase IDs CG10128-PA) or *Aedes aegypti* Nix (a distant homolog of *D. melanogaster tra2*; GenBank accession AHW46195.1) protein sequences used as query. The phylogenetic distribution of the *fle* orthologs was evaluated through a tBLASTn search against the NCBI whole-genome shotgun contigs (wgs) database using as query the amino acid translation of *fle*. The search for a putative *fle* paralog was conducted using a local BLASTp search against a database of the *A. gambiae* AgamP4.12 peptides downloaded from Vectorbase, and for other *Anopheles* species by implementing BLASTp search against a protein database for a respective species in Vectorbase. Structure of the proteins was derived by searches of the Conserved Domain Database^74^ and through structure prediction using Jpred-4.^65^ Sequence alignments were conducted using ClustalX2.^66^ Maximum likelihood phylogenetic analysis was conducted in MEGA7^67^ using JTT matrix-based model^75^ with Gamma distributed evolutionary rate differences among sites. The sequence logos were generated using WebLogo.^68^

#### dsRNA synthesis

Open reading frame fragments of the *A. gambiae* or *A. stephensi* genes were amplified through PCR from a genomic DNA template or in a one-step RT-PCR (Invitrogen) reaction from the pupal total RNA templates using gene-specific primers flanked at their 5′ ends with the T7 promoter sequence (for details on primers see Table S2). The resulting products, cloned into pGEM T-easy vector (Promega), were used directly, or after reamplification with the same primer pairs, as a template to synthesize double-stranded RNA (dsRNA) using the MEGAscript RNAi T7 kit (Life Technologies) according to manufacturer’s recommendation. Similarly, a fragment of β-lactamase (*bla*) gene was amplified by PCR from the pGEM-T Easy vector using *bla*-specific primers, each containing the T7 promoter sequence at the 5' end, and dsRNA was synthesized as described above.

#### Transfection experiments

Prior to transfection, the *A. gambiae* Sua5.1 cells were split into new culture flasks and transfection experiments were performed when cells’ confluency reached 60%–80%. Approximately 1 × 10^6^ cells per well were seeded onto 24 well plates and transfected in suspension, using 3 μl of Lipofectamine 2000 transfection reagent (Life Technologies) and 1.5 μg of dsRNA of a tested gene per well. In parallel, cells in a separate set of wells were transfected with a plasmid (0.3 μg per well) containing the eGFP open reading frame under the control of *A. gambiae* polyubiquitin promoter as a control of transfection efficiency. In addition, non-transfected control cells were cultured in a set of wells in each experiment. After approximately 24 hours, the transfection efficiency was evaluated using fluorescence microscopy. If at least 30% of the plasmid control cells per well were GFP-positive on a given plate, experimental and non-transfected control cells from that plate were harvested 48 hours post transfection to isolate total RNA with PureLink Micro kit (Life Technologies). Transfection experiments were repeated 3 times.

#### Analysis of sex-specific splicing

The effect of knockdown of the analyzed genes on the pattern of the *dsx* splicing was evaluated through RT-PCR using total RNA templates from the transfected Sua5.1 cells and primers dsxF2 and dsxR5m. Similarly, RT-PCR was used to analyze the effect of a stable *fle* knockdown on the splicing pattern of *dsx* and *fru* (the latter using primers Aga_fruF and Aga_fruR) in pupae of selected transgenic *A. gambiae* lines.

#### Transient gene silencing

Transient gene silencing experiments were conducted as described earlier.^21^ Briefly, early preblastoderm *A. gambiae* or *A. stephensi* embryos of unknown sex were microinjected with a solution containing dsRNA (1-1.5 μg/μl) of either *fle* or *bla* (as a control) gene and a plasmid (0.2 μg/μl) with the GFP gene downstream of the *Drosophila melanogaster actin 5C* promoter, or with plasmid alone as a control. Surviving first instar larvae were screened for the presence of GFP marker in the midgut cells using an M165 FC microscope equipped with a GFP filter. The larvae were sorted into GFP-negative and GFP-positive groups (*cf.* Figure S9 in Krzywinska et al.^21^). After pupation, sex of individuals was determined based on morphological characters. The experiments in *A. gambiae* were repeated three times with each of the two dsRNAs (*cf.* Figure 2A), and in *A. stephensi* were repeated two times.

#### Transgenic construct plasmid and transgenesis

To create the transgenic construct we used the p165 plasmid^61^ backbone by digesting the plasmid with MluI and NotI and ligating a linker containing the SfiI site and compatible MluI/NotI ends. The resulting plasmid was digested with SfiI and NotI, and two *in vitro* synthesized inserts were incorporated in a single ligation reaction. One insert, with SfiI and NheI ends, encoded the puromycin resistance gene *pac* under the control of the AGAP004395 promoter; the other insert, with NheI/NotI ends, encoded SV40 terminator, followed by GFP under the control of the AGAP003430 promoter (both promoters drive expression in the Sua5.1 cells, and the AGAP003430 promoter *in vivo* throughout development in gastric caeca, anterior and posterior stomach, Malpighian tubules and rectum, and at lower level in the brain, thoracic muscles and anal papillae). The resulting plasmid was digested with MluI and SfiI to clone a PCR-generated *A. gambiae* polyubiquitin promoter^76^ with MluI/BsaI-FseI ends and a PCR-generated SV40 terminator with FseI/SfiI ends. Finally, that plasmid was digested with BsaI and FseI to clone a *fle_3miR* fragment encoding a polycistronic transcript designed to silence expression of *fle*. The *fle_3miR* fragment contained three microRNAs targeting *fle* and was constructed by annealing overlapping oligonucleotides (Table S2) and PCR amplification, as previously described.^77^ After cloning into pUAST-attB vector,^62^ the *fle_3miR* fragment was released using BsaI and FseI. Thus engineered construct was excised using MluI and AsiSI and cloned into a MluI and AsiSI-cut p165-based plasmid backbone flanked by piggyBac arms and a φC31 *attB* site added to each end in opposite orientation, to create a transformation plasmid pBac_attBs_fle-3miR (GenBank: MW147152).

Early preblastoderm *A. gambiae* embryos were microinjected with a solution of pBac_pattPs_fle_3miR (0.4 μg/μl) and a helper plasmid pENTR R4-vas2-Transposase-R3 (0.2 μg/μl), containing piggy-Bac transposase reading frame under the control of the vas2 regulatory sequences,^63^ following previously described methods.^78^ Injection of approximately 1200 embryos yielded 252 G0 larvae, of which 108 individuals, that exhibited a transient fluorescent marker expression, reached the pupal stage. The emerging adults (59 males and 49 females) were placed in 9 same-sex pools for crosses with the wild-type G3 strain mosquitoes. Over 120 transgenic G1 mosquitoes were recovered from four pools of male founders, whereas no transgenic G1 individuals were produced by the female founders. Selected G1 males originating from different founder cages or exhibiting different intensity of fluorescent marker expression (if originating from the same cage; Figure 2I) were crossed to wild-type females to establish 7 independent lines. Progeny from these crosses were screened for inheritance of fluorescent marker to evaluate transgene copy number. In three lines approximately 50% of G2 individuals were transgenic, indicative of single insertions. Four other lines exhibited 60%–89% transgene inheritance, with transgenic G2 individuals representing up to three discernible classes of fluorescence intensity or pattern per line, indicative of multiple insertions. In an effort to isolate single insertion sub-lines, males derived from multiple-insertion lines and representing different fluorescence classes were backcrossed with wild-type females at consecutive generations and the number of fluorescence phenotypes was monitored at each generation. Sub-lines that, after 6 generations, produced more than one fluorescence phenotype, or in which more than 50% of the individuals inherited the transgene, were eliminated. Finally, molecular characterization was used to confirm that each line from the final set possessed a single, unique genomic transgene integration site.

No efforts have been made to generate *fle* loss-of-function mutants in this study out of feasibility concerns. Tra2 in *Drosophila*, in addition to female sex determination, is necessary for male germline development – loss of Tra2 function leads to male sterility.^79^ Fle may perform a similar role during the *Anopheles* spermatogenesis, which, combined with an apparent *fle* haploinsufficiency in females, could make recovering *fle* knockout mutants biologically impossible.

#### Identification of transgene insertion sites

The integration sites of the piggyBac element within the genome has been identified using the splinkerette PCR protocol^80^ or by inverse PCR. DNA isolated from individual pupae was used for both approaches. For inverse PCR, the DNA was digested with CviQI, HaeIII, MspI, Sau3AI, or TaqI (NEB), circularized by ligation, and amplified by PCR using primers ITRL1F and ITRL1R for piggyBac left arm, or ITRR1F^81^ and InpBacR2R for piggyBac right arm (Table S2) to isolate flanking genomic regions. The products containing genomic sequences flanking the piggyBac elements were sequenced directly, or after cloning, and genomic location of the integration sites was identified by BLAST search.

#### Abdominal musculature

Adult mosquito abdomens were dissected in phosphate-buffered saline (PBS) to release tergites with the associated musculature. The tissues were fixed in PBS containing 4% paraformaldehyde for 15 min, washed three times for 5 min in PBS, and incubated in ActinGreen 488 ReadyProbes Reagent containing AlexaFluor 488-conjugated phalloidin. After three short washes the tissues were mounted on slides and photographed with a Leica DFC365 FX camera mounted on a Leica M165 FC microscope equipped with a GFP filter. Images were processed with ImageJ.^69^

#### Real-time PCR

Total RNA was extracted from individual *A. gambiae* pupae using PureLink RNA Micro Kit (Invitrogen) according to manufacturer’s recommendations. For each sample, 500 ng of total RNA was used to synthesize cDNA with LunaScript RT SuperMix Kit (NEB). Quantitative PCR was conducted using primer pairs JK1051/JK1052 and JK1053/JK1054 to amplify, respectively, a fragment of *fle* and of the housekeeping gene encoding ribosomal protein *S7* (*rpS7*, AGAP010592) used to normalize the expression. QuantStudio 3 Real-Time PCR System (Applied Biosystems) was employed to run the reactions using Luna Universal qPCR Master Mix (NEB) at annealing temperature of 59°C. Expression levels were calculated using 2^−ΔΔCt^ method,^82^ with triple technical and three biological replicates for each sample, and all data normalized to the relative *fle*/*rpS7* expression in the samples of the wild-type female pupae.

#### RNA-seq analysis

Total RNA was extracted using the Trizol method and quality-checked using TapeStation (Agilent). Triplicate samples of female pupae from wild-type G3 line and from transgenic 4M4B line were used for transcriptome sequencing. The TruSeq library preparation protocol (Illumina) was followed by 150 bp paired-end sequencing using NovaSeq 6000 sequencing system (Illumina). The reads were pseudo-aligned to the *A. gambiae* transcriptome genebuild AgamP4.12 using Kallisto v0.46.^70^ Transcripts per kilobase million (TPM) value was quantified for each transcript and averaged across multiple replicates of the same sample. As a further check of statistical robustness, differential expression analysis was performed using DESeq2,^71^ with filtering out transcripts covered by less than 10 reads among all samples, and then shrinking log2-fold changes using apeGLM.^72^

### QUANTIFICATION AND STATISTICAL ANALYSIS

For the transient *fle* experiments, the probability of the observed microinjection results under the null hypothesis that there is no sex bias difference between the GFP-positive and GFP-negative (or control) groups was calculated using Fisher’s exact test. For the real-time PCR experiments, Student’s t test was used to evaluate statistical differences between the relative *fle* expression levels in the wild-type and transgenic strains after performing a goodness of fit test.

## Supplemental Information

Document S1. Figures S1–S3 and Tables S1 and S2

Document S2. Article plus Supplemental Information
