# Supplementary material for: femaleless Controls Sex Determination and Dosage Compensation Pathways in Females of Anopheles Mosquitoes
Source: Curr Biol. 2021 Mar 8;31(5):1084–1091.e4. doi: 10.1016/j.cub.2020.12.014 (PMC7955153; doi:10.1016/j.cub.2020.12.014)
Supplement: Document S1. Figures S1–S3 and Tables S1 and S2 [file mmc1.pdf]

Current Biology, Volume *31*

Supplemental Information

femaleless Controls Sex Determination and Dosage Compensation Pathways in Females of *Anopheles* Mosquitoes

Elzbieta Krzywinska, Luca Ferretti, Jianwei Li, Jian-Chiuan Li, Chun-Hong Chen, and Jaroslaw Krzywinski

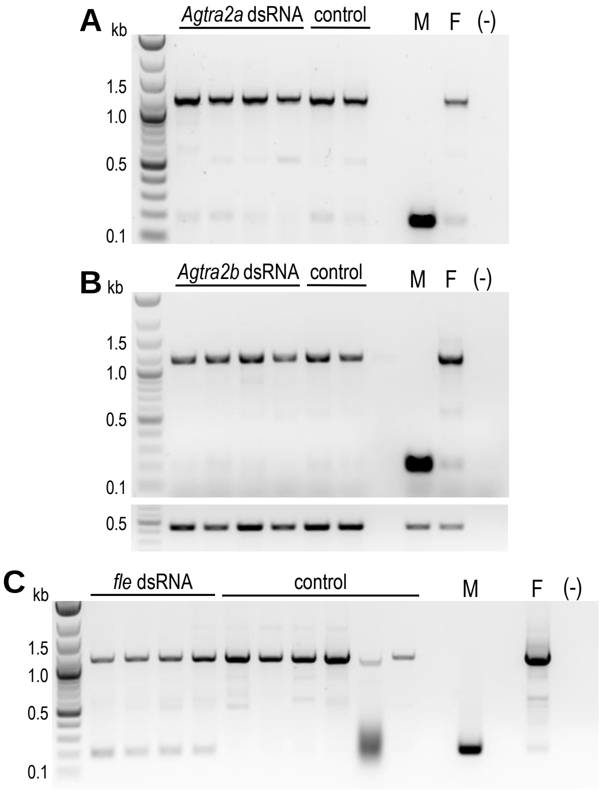

Figure S1. Homologs of *tra2* do not control sex determination in *A. gambiae.* Related to Figure 1. RT-PCR analysis of *dsx* splicing in the *A. gambiae* Sua5.1 cells transfected with in the vitro–synthesized dsRNA of *A. gambiae tra2a* (AGAP029421) (**A**), *tra2b* (AGAP006798) (**B**), and *fle* (AGAP013051) (**C**), as compared to the control cells. No difference in *dsx* splicing pattern between the dsRNA-transfected and the control cell samples in **A** or **B** indicates that neither of the *tra2 paralogs* is involved in the *A. gambiae* sex determination pathway. In contrast, *fle* dsRNA-transfected cells produce fewer female-specific *dsx* transcripts, which coincides with a clear production of male *dsx* transcript forms (**C**). Cropped elements of the gel image presented in **C**, with two poor quality RNA control sample lanes omitted, are shown in the main body of the paper as Fig. 1A. M and F, male and female pupae; (-), negative control.

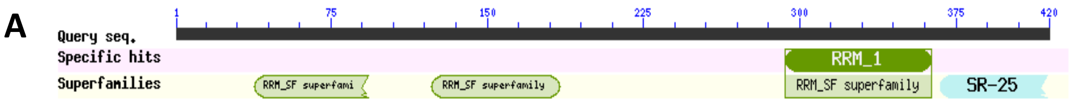

Name	Accession	Description	Interval	E-value
RRM_1	pfam00076	RNA recognition motif. (a.k.a. RRM, RBD, or RNP domain)	293-363	2.88e-15
SR-25 superfamily	cl26281	Nuclear RNA-splicing-associated protein; SR-25, also known as ADP-ribosylation factor-like factor 6-interacting protein 4	367-419	9.25e-06
RRM_SF	cl17169	RNA recognition motif (RRM) superfamily; RRM, also known as RBD (RNA binding domain) or RNP (ribonucleoprotein domain)	123-185	6.34e-05
RRM_SF	cl17169	RNA recognition motif (RRM) superfamily; RRM, also known as RBD (RNA binding domain) or RNP (ribonucleoprotein domain)	38-93	5.89e-04

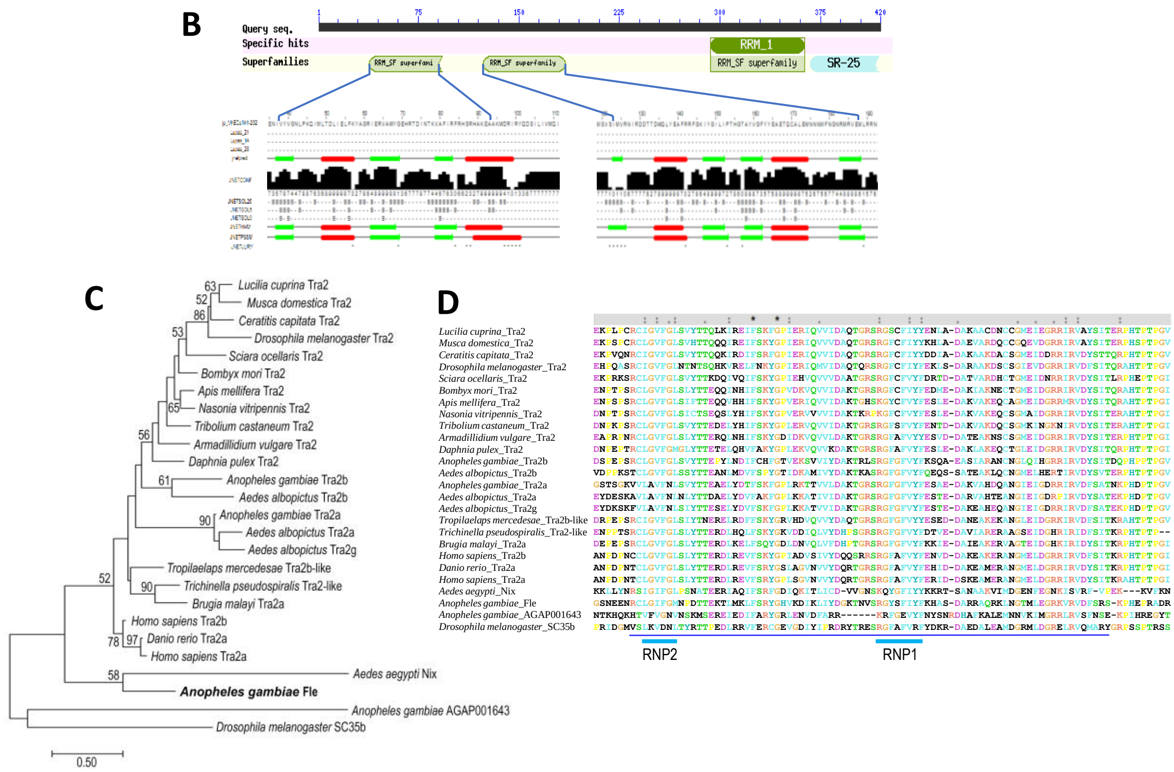

Figure S2. *A. gambiae* Fle is an RRM protein with putative binding sites in *doublesex* and *fruitless*, and is not closely related to TRA2*.* Related to Figure 1. (**A**) Conserved domains in the *A. gambiae* Fle protein identified through a search against the Pfam v.31.0 database. (**B**) Jpred4-based secondary structure prediction of the putative RRM regions in Fle. α-helices are marked in red and β-sheets in green. The truncated first putative RRM lacks the β_4 sheet. (**C**) The maximum likelihood phylogenetic tree of Fle, representative Tra2, and other RRM proteins inferred with MEGA v. 7.0.26 using the JTT matrix-based model and a discrete Gamma distribution to model evolutionary rate differences among sites. The tree is drawn to scale, with branch lengths measured in the number of substitutions per site.The analysed data set consisted of amino acid sequences aligned using ClustalX2. All gapped alignment positions were eliminated prior to analysis; there were 82 positions in the final data set, covering RRM (C-terminus proximal in case of Fle) and its immediate flanks (see **D**). The tree was rooted with the *D. melanogaster* SR family splicing factor SC35. Numbers above or below branches representing bootstrap support >50% are based on 500 ML bootstrap replications. (**D**) An alignment of the amino acid region used for the phylogenetic reconstruction. Identical/similar positions are marked above, and the location of the RRM (thin line) and RNP sequence elements below, the alignment.

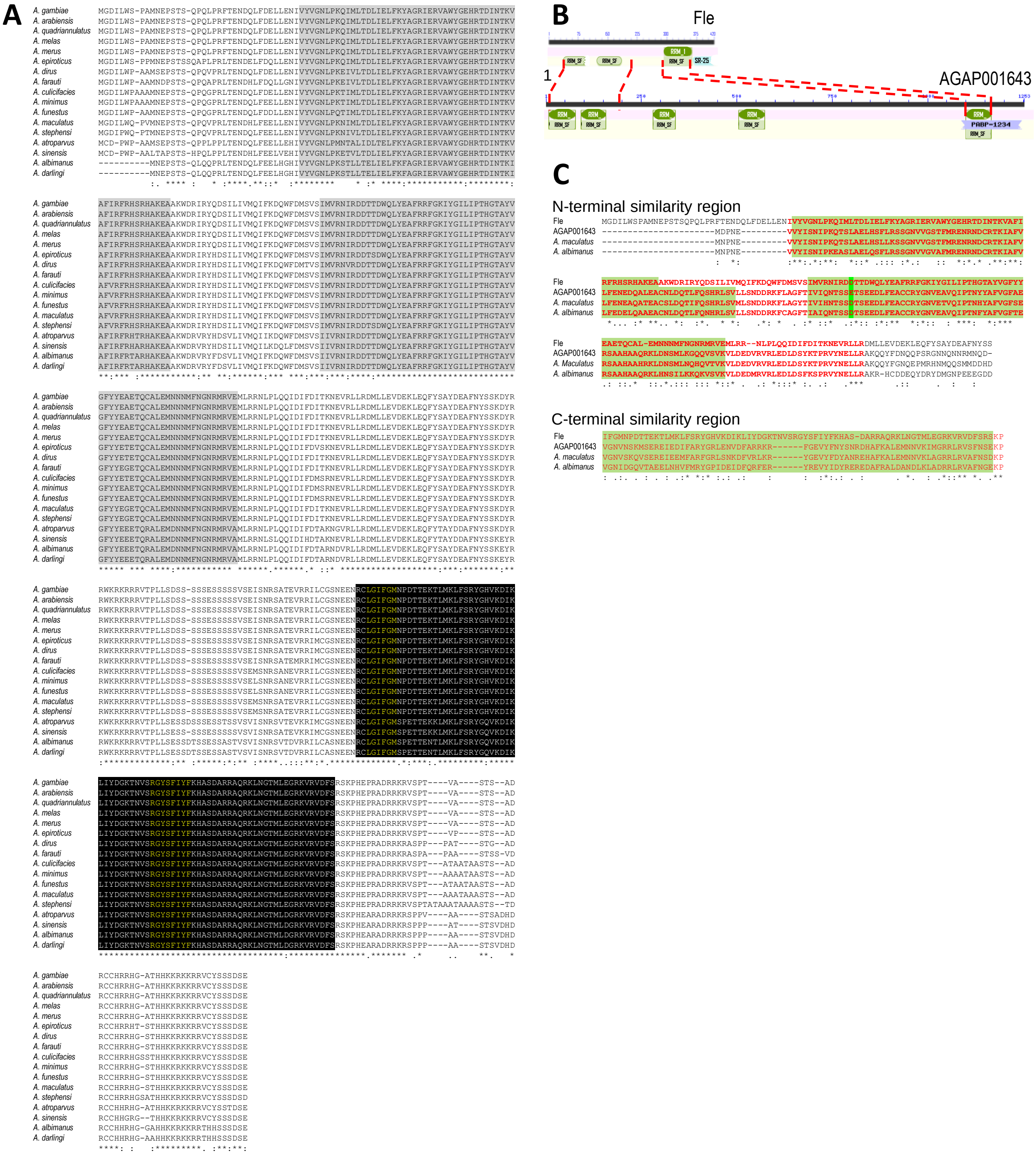

Figure S3. A highly conserved *fle* may have originated by gene duplication in the recent common ancestor of *Anopheles.* Related to Figure 1. (**A**) Amino acid sequence alignment of the *fle* orthologs from *Anopheles* species. The RRM region showing similarity to Tra2 RRM is shaded black, with the RPN-2 and RPN-1 elements in yellow font. Two other putative RRM regions are shaded grey. (**B**) A comparison of the Fle and AGAP001643 protein structures. Boundaries of a similarity region (BLASTp *e*-value = 4e-20) are marked by red lines. (**C**) Amino acid alignment of the N-terminal and C-terminal part of Fle and AGAP001643, respectively, with the similarity region marked in red, the RRMs shaded green, and the codon interrupted by an intron shaded bright green. Note a higher sequence similarity within the N-terminal region. AGAP001643 orthologs from *A. maculatus* and *A. albimanus* that split from *A. gambiae* approximately 36-70 Mya and 100 Mya, respectively, are also shown. The phylogenetic tree shown in Figure S2C has been inferred using a slightly extended C-terminal region (cf. Figure S2D) than that presented in (**C**).

Line	pBAC arm	Flanking genomic sequence	Fragment length	% Identity to genome	Chromosomal integration position
3M	L	**TTAA**TAGGCACGTGCCTTCG	171	99	3R: 15803701
	R	CCTGCCTGCTCGAGCT**TTAA**			3R: 15803704
4M2B	L only	**TTAA**AGGCTGTGGTACACTT	294	100[a]	UNK NW_161049: 12
4M3	L	**TTAA**GGGTCTGCGCCAGGGG	224	89 [b]	Undetermined
	R	AGGCTTTTTGCTACCA**TTAA**	248	91 [b]	Undetermined
4M4B	L	**TTAA**ATCACCTTGTAACGAT	262	99	2R: 21049616
	R	TTGATTCTTGTTAGCT**TTAA**	304	98	2R: 21049618
4M4C	L	**TTAA**ACTCAACGATGGCAAT	120	98, 95[c]	3R: 28923242
	R	TGTCAGGCGGTGGCATTTAA	180	98	3R: 28923245
4M6	L	**TTAA**AAGAGGTGAGCCCAGA	459	99	3L: 40448623
	R	ATAATCCATCAACTGT**TTAA**	169	99	3L: 40448620

Table S1. Transgene integration sites in the *A. gambiae* lines generated in this study. Related to Figure 2.

[a] Integration site within the retrotransposon *zanzibar* fragment of unknown chromosomal location; maximum 93% identity within the mapped *An. gambiae* PEST scaffolds.

[b] Hit to a highly variable repetitive region differing between the *A. gambiae* strains; 94% identity to the *A. colluzzii* strain M genome.

[c] The sequence of a fragment isolated adjacent to the pBac L arm yielded a gapped BLAST result, due to the presence of a Tsessebe transposon insertion in the corresponding region within the *A. gambiae* PEST genome. Ungapped alignments, indicating absence of the Tsessebe insertion, were obtained with four other *A. gambiae* genome assemblies available in the NCBI whole genome shotgun contigs database.

Target/Name	Name/sequence (5’ – 3’)	
	**PCR primers**	
A. gambiae fle (AGAP013051)	T7-fle-F2<br>TAATACGACTCACTATAGGGTGCGCACTGGAAATGAACAA	T7-fle-R2<br>TAATACGACTCACTATAGGGGTTCGTTTTGCCGTCGTAGA
	T7-fle-F2	T7-fle-R3
	Sequence as above	TAATACGACTCACTATAGGGACAGCGGTTCTCCTCGTTC
A. stephensi fle (ASTE008781)	Aste_fleT7F<br>TAATACGACTCACTATAGGGATATACGGCATCCTGATACC	Aste_fleT7R<br>TAATACGACTCACTATAGGGGCTTAAAGTAGATGAACGAG
tra2a (AGAP006798)	T7_Ag6798-F<br>TAATACGACTCACTATAGGGTGCAGTGAAGTGTTGTTATTTTG	T7_Ag6798-R<br>TAATACGACTCACTATAGGGCTTTCGCAGTGGACCAAACT
tra2b (AGAP029421)	T7_Agtra2b-F<br>TAATACGACTCACTATAGGGTCCAGCAGCCATTGTGTCTA	T7_Agtra2b-R<br>TAATACGACTCACTATAGGGACTACCGACTTTTCCACCGT
dsx (AGAP004050)	dsxF2 CCAGAACCTGTAAATCTCCTAC	dsxR5m GATGACTTCACCACCGCTTC
fru (AGAP000080)	Aga_fruF GGCAGCATCGGACTTGTTCA	Aga_fruR ACGTCGCACAGTTTCTCATC
rpS7 (AGAP010592)	S7F TGCTGCAAACTTCGGCTAT	S7R CGCTATGGTGTTCGGTTCC
bla	blaF_T7<br>TAATACGACTCACTATAGGGAACTTTATCCGCCTCCATCC	blaR_T7<br>TAATACGACTCACTATAGGGGCTATGTGGCGCGGTATTAT
A. gambiae fle RT_q-PCR	JK1051 CGGATACGCTACCAAGACTCAATAC	JK1052 GTCGGTTGTATCGTCGCGT
rpS7 RT_q-PCR	JK1053<br>CAACAACAAGAAGGCGATCATCATC	JK1054 GCGCTCGGCAATGAACAC
Inverse PCR pBac right arm	ITRR1F TACGCATGATTATCTTTAACGTA	InpBacR2R TTGCTACTGACATTATGGCT
	**Oligonucleotides for microRNA cistron construction**	
Mir6.1_5’EcoRI/BsaI/BglII	GGCGAATTCATGGTCTCTTATGAGATCTTTTAAAGTCCACAACTCATCAAGGAAAATGAAAGTCAAAGTTGGCAGCTTACTTAAACTTA	
Mir6.1_3’NotI/FseI/BamHI	GGCCGCGGCCGCTATGGCCGGCCGGATCCAAAACGGCATGGTTATTCGTGTGCCAAAAAAAAAAAAAATTAAATAATGATGTTAGGCAC	
AGAP013051_miR-1-1	GGCAGCTTACTTAAACTTAATCACAGCCTTTAATGTGCATCGTACAGACATCAACACGTAAGTTAATATACCATATC	
AGAP013051_miR-1-2	AATAATGATGTTAGGCACTTTAGGTACGCATCGTACAGACATCAACACGTAGATATGGTATATTAACTTACGTG	
AGAP013051_miR-2-1	GGCAGCTTACTTAAACTTAATCACAGCCTTTAATGTGGATACGCTACCAAGACTCAATTAAGTTAATATACCATATC	
AGAP013051_miR-2-2	AATAATGATGTTAGGCACTTTAGGTACGGATACGCTACCAAGACTCAATTAGATATGGTATATTAACTTAATTG	
AGAP013051_miR-3-1	GGCAGCTTACTTAAACTTAATCACAGCCTTTAATGTAAAGACATCAAGCTAATCTACGTAAGTTAATATACCATATC	
AGAP013051_miR-3-2	AATAATGATGTTAGGCACTTTAGGTACAAAGACATCAAGCTAATCTACGTAGATATGGTATATTAACTTACGTA	

Table S2. Oligonucleotides used in the study. Related to STAR Methods.
